# Determinants of Copper Resistance in *Acidithiobacillus Ferrivorans* ACH Isolated from the Chilean Altiplano

**DOI:** 10.3390/genes11080844

**Published:** 2020-07-24

**Authors:** Sergio Barahona, Juan Castro-Severyn, Cristina Dorador, Claudia Saavedra, Francisco Remonsellez

**Affiliations:** 1 Laboratorio de Microbiología Aplicada y Extremófilos, Departamento de Ingeniería Química, Universidad Católica del Norte, Antofagasta 1240000, Chile; jsevereyn@gmail.com; 2 Laboratorio de Complejidad Microbiana y Ecología Funcional, Departamento de Biotecnología, Facultad de Ciencias del Mar y Recurso Biológicos, Universidad de Antofagasta, Antofagasta 1240000, Chile; cristina.dorador@uantof.cl; 3 Programa de Doctorado en Ingeniería de Procesos de Minerales, Facultad de Ingeniería, Universidad de Antofagasta, Antofagasta 1240000, Chile; 4 Centro de Biotecnología y Bioingeniería (CeBiB), Universidad de Antofagasta, Antofagasta 1240000, Chile; 5 Laboratorio de Microbiología Molecular, Facultad de Ciencias de la Vida, Universidad Andrés Bello, Santiago 8320000, Chile; csaavedra@unab.cl; 6 Centro de Investigación Tecnológica del Agua en el Desierto (CEITSAZA), Universidad Católica del Norte, Antofagasta 1240000, Chile

**Keywords:** *A. ferrivorans* ACH, copper resistance, Chilean Altiplano, bioleaching

## Abstract

The use of microorganisms in mining processes is a technology widely employed around the world. Leaching bacteria are characterized by having resistance mechanisms for several metals found in their acidic environments, some of which have been partially described in the *Acidithiobacillus* genus (mainly on *ferrooxidans* species). However, the response to copper has not been studied in the psychrotolerant *Acidithiobacillus ferrivorans* strains. Therefore, we propose to elucidate the response mechanisms of *A. ferrivorans* ACH to high copper concentrations (0–800 mM), describing its genetic repertoire and transcriptional regulation. Our results show that *A. ferrivorans* ACH can grow in up to 400 mM of copper. Moreover, we found the presence of several copper-related makers, belonging to *cop* and *cus* systems, as well as rusticyanins and periplasmatic *acop* protein in the genome. Interestingly, the ACH strain is the only one in which we find three copies of *copB* and *copZ* genes. Moreover, transcriptional expression showed an up-regulation response (*acop, copZ, cusA, rusA,* and *rusB*) to high copper concentrations. Finally, our results support the important role of these genes in *A. ferrivorans* copper stress resistance, promoting the use of the ACH strain in industrial leaching under low temperatures, which could decrease the activation times of oxidation processes and the energy costs.

## 1. Introduction

Bioleaching processes can be defined as systems in which capable microorganisms catalyze the extraction and recovery of metals from sulfide mineral ores [1]. In the last two decades, the commercial interest in this type of technology has considerably increased owing to the resulting economic benefits, as it is a globally established biotechnological technique [2]. It is well-known that leaching microorganisms are constantly exposed to acid leach solutions, containing heavy metals such as arsenic, zinc, iron, nickel, and copper, and reaching toxic concentrations for most living organisms nearby (0.1–1 M) [3].

Specifically, in copper-sulfides’ bioleaching processes, the copper concentrations are very high, up to 300 mM (19.05 g/L of Cu^2+^) [4]. Therefore, in order to thrive under leaching conditions, the microorganisms must be highly resistant to several toxic compounds. Thus, acidophilic microorganisms involved in bioleaching processes can resist copper concentration >100 mM [5]. The toxic effects of some of these compounds are related to enzymatic function blocking, transport inhibition, or cellular membrane perturbations [6]. Specifically, copper easily interacts with free radicals, specifically with molecular oxygen, generating hydroperoxide radicals. These molecules promote an oxidative stress state on the cell, which damages the cell membrane, DNA, and enzymes [6]. Therefore, to control the heavy metal concentrations, maintain cellular homeostasis, and catalyze the efficient recovery of the interest or profitable compound, leaching microorganisms must resist the implied toxicity [7].

The presence of diverse resistance mechanisms is a response to selective pressures from leach environments [8]. Acidophilic microorganisms are more tolerant of a high concentration of heavy metals compared with neutrophilic microorganisms. This capacity is provided by the combination of passive and active mechanisms, enabling them to grow in the presence of high metal concentrations [9]. The passive mechanisms correspond to all intrinsic responses such as metallic complex formation with sulfate, chemiosmotic gradient generation (internal membrane potential electro-positive), and biofilm formation on the mineral surface [9,10,11]. On the other hand, the active mechanisms include efflux pumps that use ATP to expel metal ions to the extracellular space across the membrane (e.g., Cop P-type ATPase) [12]. Additionally, other efflux pumps have been described (RND systems) that are proton-gradient-dependent and allow for pumping not only metals ions, but also drugs, organic solvents, and fatty acid, among other compounds [13]. Finally, some leaching microorganisms can accumulate large quantities of cytoplasmic polyP granules that can inactivate heavy metals by sequestration [14].

One of the most studied bioleaching microorganisms, which is usually present in many biomining processes, is *Acidithiobacillus ferrooxidans* [15]. Hence, many studies describe the ability of this genus to grow in high copper concentrations [5,9,16,17,18]. For example, the capacity of *A. ferrooxidans* ATCC 23270 to survive in high copper concentrations has been associated with the presence of at least ten genes, namely, *copA1*, *copA2*, *copB,* and *cusCBA* (RND system) [19]. In addition, the presence of some copper chaperones such as *cusF* and *copC* has been associated with resistance [8,20]. Among the *Acidithiobacillus* genus, the *A. ferrivorans* species was initially described as the unique psychrotolerant member, characterized by its ability to oxidize ferrous iron and sulfur, as well as to oxidize inorganic sulfur compounds and sulphide minerals [21]. To date, only five strains (SS3, CF27, ACH, PQ33, and YL15) have been described for this species, isolated from several countries around the world (Russia, USA, China, Peru, and Chile) [21,22,23,24,25]. However, *A. ferrivorans*’ ability to tolerate high heavy metal concentrations remains mostly unexplored, mainly because its psychrotolerant capacity draws greater research interest. Nevertheless, although there is information about genetic determinants for heavy metal resistance in *A. ferrivorans*, most research has focused only on identifying the presence/absence of genes potentially involved in these processes [23,26,27]. In this case, several genes involved in copper resistance (including RND and Cop systems) have been identified in the SS3, CF27, and YL15 genomes [23,26,27]. However, the function and capacity of those genes to yield the copper-resistant phenotype has not yet been demonstrated, nor has it been correlated with processes at low temperatures.

Therefore, understanding these functions and physiology could be helpful in their usage or application in different industrial scenarios, as there are several known advantages of using bacteria capable of leaching sulphide minerals with high copper content at low temperatures. First, the processes at low temperature can mainly save energy (compared with high temperature processes); second, they could accelerate bioleaching processes in the early stages of mineral oxidation (which will also reduce the operation time to obtain copper). Specifically, in Chile, most bioleaching operations and mining wastes are located in the Andes (high altitude), where mean temperatures are usually −5 °C or less [28,29]. On the other hand, low temperatures have a direct effect on mineral oxidation rates, as mesophilic microorganisms are unable to generate ferric ion (Fe^3+^) in this condition, which is the main leaching factor in these processes [30]. Given the previous background, we aimed to understand the copper response mechanisms used by the *A. ferrivorans* ACH strain isolated from a polyextremophilic environment (Chilean Altiplano) to resist high metal concentrations. For this, we described the copper resistance genetic repertoire and determined the effect of high copper concentrations on growth capacity and transcriptional expression.

## 2. Materials and Methods 

### 2.1. Bacterial Strain and Growth Conditions

Previously, our group isolated *A. ferrivorans* ACH from a shallow acid stream (pH < 3) located in the Chilean Altiplano (Cerro Aroma River—Tarapacá Region) [25]. ACH strain cells were grown at 28 °C and 10 °C in the absence or presence of copper sulfate in 9 K medium (1 g/L (NH_4_)_2_SO_4_, 0.5 g/L MgSO_4_ × 7H_2_O, 0.5 g/L K_2_HPO_4_, 0.1 g/L KCl, and 33 g/L Fe_2_SO_4_ × 7 H_2_O as a unique energy source) [25]. The pH was adjusted at 1.7 with sulfuric acid. Microbial growth was monitored by counting the unstained cells number through a Neubauer chamber under a phase-contrast microscope (Olympus, CX21) in triplicate. Successive subcultures were made with increasing CuSO_4_ concentrations (100, 200, 300, 400, and 800 mM) for adaptation (in triplicate). The cultures used for expression assays were prepared in 1 L Erlenmeyer flasks with orbital agitation at 120 rpm containing 800 mL of media.

### 2.2. Search for Copper Resistance Genetic Determinants in the ACH Strain Genome

A list with known and described copper response proteins for the *Acidithiobacillus* reference strains was made from UniProt (Table S1). These markers were queried using BLAST [31] against the ACH strain genome (GenBank accession JAAZUD000000000 (BioProject: PRJNA624122); Table S2) to determine the presence, copy number, and identity level. Moreover, we used several other available web tools, such as InterPro Scan [32], T-coffe [33], CDD/SPARCLE [34], and Metal Detector [35], to make the alignments and check the protein functions. Moreover, to visualize the genetic contexts of the interest markers, we used Genious^®^ 10.2.2 software [36]. For comparisons, we used the GenBank available *A. ferrivorans* genomes (Table S3).

### 2.3. Total RNA Extraction and cDNA Synthesis from Copper-Cultured A. Ferrivorans ACH

For gene expression assays, the ACH cells were grown in the absence of copper (control) and in the presence of three CuSO_4_ concentrations (200 mM, 300 mM, and 400 mM) until the late exponential growth phase was reached. Three biological replicates were made for each experimental condition. Then, total RNA extraction was carried out using a previously reported protocol [37,38] modified by the use of TRIzol (Invitrogen). Then, RNA integrity was verified using 1% agarose electrophoresis, and the remaining DNA was eliminated by 1 U of RQ1 RNase-Free DNase (Promega) following the manufacturer’s instructions. Next, quantification was carried out using the Qubit RNA HS assay kit (Thermo Fisher). Finally, for cDNA synthesis, 1 µg of total RNA was reverse transcribed using the ImProm-II (Promega) system following the manufacturer’s instructions.

### 2.4. Relative Gene Expression Quantification

Specific internal primers for our interest genes were designed using the Primer3 software [39], with specificity and non-dimerization checks (Table S4). The PCR reaction was carried out as follows: 10 min at 95 °C followed by 40 cycles of 5 s at 95 °C and 20 s at 60 °C. Transcript levels were quantified using the Fast SYBR Green Master Mix (Applied Biosystems™) on a StepOne™ Real-Time PCR system (Applied Biosystems™). Gene expression levels were calculated according to Pfaffl [40] using 16 S rRNA gene expression for normalization. Three independent biological experiments were carried out, with three technical replicates each. Statistical significance was determined using an unpaired t-test with a 95% confidence interval (two-tailed *p*-value).

## 3. Results and Discussion 

### 3.1. Effects of Copper on ACH Strain Growth

**Figure 1:**
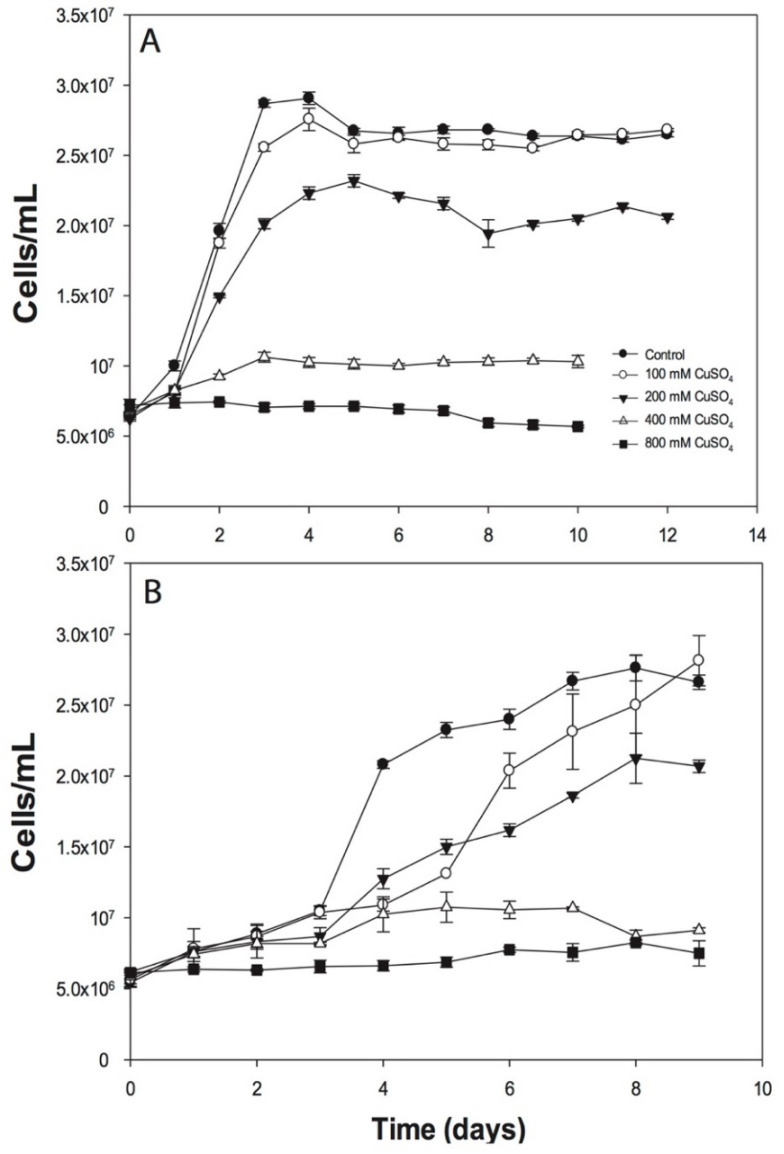
Growth of *A. ferrivorans* ACH in the presence of different CuSO_4_ concentrations. Cells were incubated at 28 °C (**A**) and 10 °C (**B**). The mean values of three independent biological experiments (with three technical replicates each) were plotted.

The *A. ferrivorans* ACH cells were adapted to grow at different CuSO_4_ concentrations (0–800 mM) and growth rates decreased when copper concentrations were above 100 mM (Figure 1). Moreover, the ACH strain was able to grow with the addition of 100 mM CuSO_4_ similarly to the control (culture in the absence of copper), reaching around 2.7 × 10^7^ cells/mL in about four days. Then, the growth decreased until the stationary phase was reached, with 2.6 × 10^7^ cells/mL (Figure 1A). In addition, the 200 mM CuSO_4_ condition appears to generate a negative effect on cell growth, decreasing the number to 2.3 × 10^7^ cells/mL after six incubation days. Moreover, the microorganisms were drastically affected when the cells were grown at 400 and 800 mM of CuSO_4_ (Figure 1A). Additionally, similar results were obtained when the microorganisms were grown at 10 °C. Nevertheless, for this temperature, the cell numbers were lower as compared with those observed for the 28 °C condition; in addition, there was a longer lag phase (Figure 1B).

As mentioned previously, the bioleaching microorganisms are resistant to high heavy metal concentrations in solution. Although some *Acidithiobacillus* genus members resist CuSO_4_ ranges of 40–100 mM, this capacity is poorly understood in the *A. ferrivorans* species. For instance, Hallberg and collaborators reported that the *A. ferrivorans* strains NO-37, CF27, Peru6, and OP14 were able to resist copper concentrations close to 50 mM [21]. Moreover, the *A. ferrivorans* YL15 strain was reported to grow in the presence of up to 400 mM of CuSO_4_ [23], which we also observed in the ACH strain. Although its growth rate decreases, it can survive at this concentration (Figure 1). Interestingly, both strains were isolated from highland arid environments located in China (4600 m.a.s.l.) [23] and Chile (4200 m.a.s.l), respectively [25]. Furthermore, the increase in growth times for *Acidithiobacillus* members suggests that respiration is affected, and iron oxidation is delayed owing to the presence of copper [41]. Additionally, the effects of low temperature in the cells are well-known, mainly impacting the cytoplasm and membrane fluidity, thus preventing the correct nutrient/ion flow and enzymatic activity, and also increasing the microorganism growth times [42]—a phenomenon observed in our assays (Figure 1B).

### 3.2. Genetic Determinants of Copper Resistance in ACH Strain

**Figure 2:**
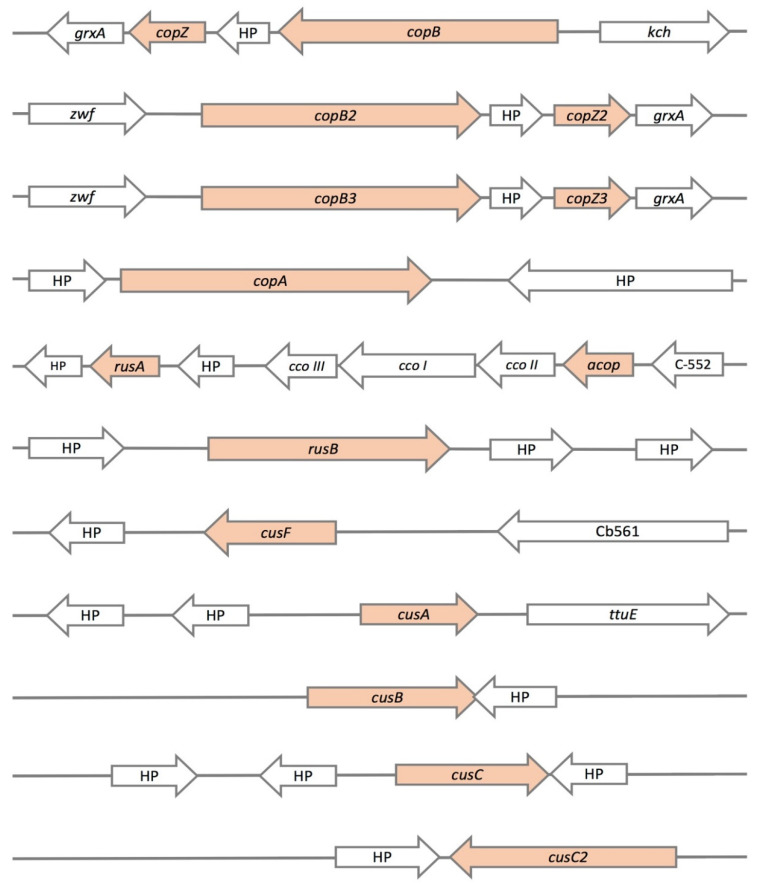
Presence and genomic context of copper resistance genes found on the *A. ferrivorans* ACH genome. *copZ*: metallochaperone; HP: hypothetical protein; *copB*: ATPase P-type; *kch*: potassium channel; *zwf*: glucose-6-phosphate-dehydrogenase; *grxA*: glutaredoxin; *copA*: ATPase P-type; *rusA*: rusticyanin A; cco III: cytochrome c oxidase polypeptide III; cco I: cytochrome c oxidase polypeptide I; cco II: cytochrome c oxidase polypeptide II; *acop*: cupredoxin; C-552: cytochrome c 552; *rusB*: rusticyanin B; *cusF*: periplasmic metallochaperone; cb561: cytochrome b561; *cusA*: inner membrane transporter (RND family); *ttuE*: Pyruvate kinase; *cusB*: periplasmic adaptor factor protein (membrane fusion protein (MFP) family); *cusC*: outer membrane factor (OMF).

In the genome of the *A. ferrivorans* ACH strain, we found the sequences of several copper resistance genes (Table 1). Moreover, Figure 2 shows the genomic contexts of all detected genes based on their sequence identity regarding reference proteins. Among these, we found several Cu-ATPase pumps (3 copies of *copB*; *copA*), RND system (*cusA*, *cusB,* and two copies of *cusC*), metallochaperones (*cusF* and three copies of *copZ*), Rusticyanin (*rusA* and *rusB*), and the periplasmic Acop protein (*acop*). Moreover, we have to mention the presence of six *cusCBA*-like gene clusters scattered in the ACH genome (Figure S1).

### 3.3. Comparison of Copper Resistance Genes Identified in A. Ferrivorans Strains

**Figure 3:**
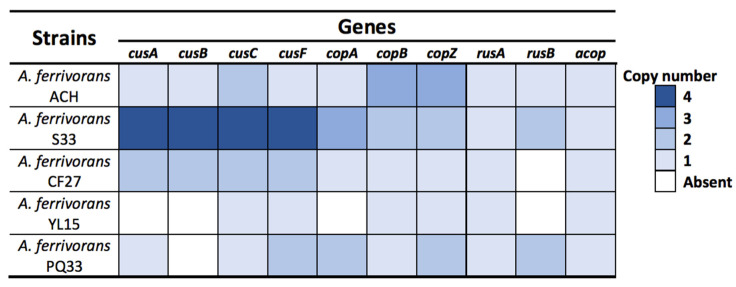
Presence/absence of copper genomic determinants in the *A. ferrivorans* species. The heat scale shows the copy numbers of each gene present in the genome of all *A. ferrivorans* strains.

*A. ferrivorans* is considered the only *Acidithiobacillus* genus member capable of growing at low temperatures, being the first reported acidophilic psychrotolerant [21,30]. In addition to the ACH strain, only four other strains have been described in the literature: SS3 (Russia), CF27 (USA), YL15 (China), and PQ33 (Peru) [21,22,23,24,25]. The genomes of all five *A. ferrivorans* strains (available in GenBank: Table S3) were searched and compared for the presence of copper resistance genes. Among those, the RND-system (*cus*), Cu-ATPases pump (*cop*), metallochaperones (*cusF* and *copZ*), rusticyanin (*rus*), and “acidophile cytochrome c partner” (*acop*) were mostly found in all the genomes, although all five genetic repertoires were different in number and composition (Figure 3). Furthermore, the most notable differences between them were related mainly to copy number. Nevertheless, some differences were found regarding the absence of certain genes, such as *cusCBA* in the YL15 and PQ33 strains. Furthermore, as we can see in Figure 3, there is some pattern showing a modest correlation between a greater resistance level in the strains that has a broader gene repertoire (SS3 and ACH). However, this does not fully explain the presented capacity of each strain and we cannot rule out the action of other factors not yet identified.

Gonzalez and collaborators previously reported the presence of eleven gene clusters potentially involved in copper resistance in the *A. ferrivorans* SS3 genome—specifically, *cusCBA-hyaC-cusF* (Acife_0498–0502 (Figure S2A); Acife_0810–0814 (Figure S2B); Acife_0894–0898 (Figure S2C)), *cusCBA* (Acife_2145–2147) (Figure S2D), and *cusCBA-like* (Acife_0050–0052; Acife_0198–0200; Acife_0822–0824; Acife_1415–1417; Acife_2127–2129; Acife_2140–2142; Acife_2417–2419) clusters (Table S5) [26]. In this context, using the SS3 sequences as a reference, we compared the amino acid identity of these markers with those found in *A. ferrivorans* ACH, as well as with the other *A. ferrivorans* genomes. Our results showed that *A. ferrivorans* ACH shared a high similarity with only three of the SS3 *cus* markers—specifically, *cusF3* (Acife_0502) (100%) (Figure S2A), *cusB* (Acife_0811) (98%) (Figure S2B), and *cusC* (Acife_2145) (100% and 97%) (Figure S2D). On the other hand, the *cusA* gene showed low sequence similarity (30% with *cusA3* (Acife_0500) (Figure S2A). In addition, *cusF3* and *cusA3* genes have been reported as being part of the ATCC 53993 strain copper resistance [38].

Then, we identified three copies of *copB* and *copZ* genes in the ACH strain (Figure 2 and Figure 3), unlike the SS3 strain, which has only two copies of these genes (Figure S3A,D). Moreover, these additional gene copies could be the key factor conferring the high resistance level represented by the ACH strain. This makes it comparable to the SS3 strain resistance level, despite its having a smaller gene repertoire. Additionally, it has been widely described that bacteria with dynamic genomes could increase their genetic repertoires, thereby enhancing their phenotypic capacities, such as copper resistance [43,44]. On the other hand, all copies of *copB* and *copZ* genes are part of a possible operon, which also includes a *grxA* gene in the ACH strain (Figure 2). Additionally, this *grxA* gene encodes a glutaredoxin protein, which is involved in the response to the oxidative stress generated by reactive oxygen species (ROS) through the restoring of glutathione (GSH) [45]. These proteins are major thiol-disulfide oxidoreductases (containing a redox-active disulfide), which are essential to maintaining intracellular redox homeostasis [46]. As mentioned previously, the bioleaching microorganisms usually inhabit environments with acidic pH and a high concentration of copper and iron—conditions favoring ROS formation (specifically OH^−^ or O_2_^−^) through Fenton or Haber–Weiss reactions [47,48]. Nevertheless, the depletion of antioxidants like GSH in response to copper-induced oxidative stress would explain the link between these mechanisms and their organization in the identified “operons”. Clear examples of this are the presence of *arsT* and *cdr* genes within both arsenic response clusters of *Microbacterium* and *Exiguobacterium*, respectively [49,50].

Interestingly, we found that YL15 and CF27 strains have only one *rusA* gene and that no *rusB* was detected. Additionally, both SS3 and PQ33 strains have two copies of the *rusB* gene, implying a significant role in their high copper resistance. However, no expression assays can corroborate this affirmation. Additionally, the amino acid sequence of both rusticyanin genes (*rusA* and *rusB*) found in the ACH genome was identical (100%) to those from the SS3 strain (Figure S3B,C). On the other hand, Tran and collaborators reported the presence of at least seventeen genes involved in CF27 strain copper resistance (*copZ*, *copA*, *copB*, *cusCBA*-like, and putative phosphate transporter (*Pho84*)) [27]. Comparing these genes to those from the ACH strain, we found a high similarity only with *copZ* (100%) (AFERRI_420163), *cusC* (98%) (AFERRI_10103), and *copA* (99%) (AFERRI_140009). Then, Peng and collaborators described at least four *cusCBA* systems and one *cop* system (specifically, CopB protein) in the YL15 strain genome [23]. Moreover, this *copB* (BBC27_RS13630) has a high similarity to the protein found in the ACH strain (97%).

Finally, ACH and the other strains share the same *cusCBA-like* genes with a high-amino-acid-sequences identity (100% similarity in all proteins) as compared with those from the SS3 strain. However, the role and function of these have yet to be experimentally tested (Figure S1 and Table S5). It is important to consider that genetic dosage and redundancy could potentially be the reason for the differential copper resistance presented by each strain and also could be the result of niche-specific pressure as an adaptation to their environmental conditions. This has been studied in several organisms in response to different types of stress; good examples are *Deinococcus radiodurans* and *Exiguobacterium* sp. SH31 [51,52,53].

### 3.4. Conserved Amino Acid Motifs in ACH Strain Copper Resistance Proteins

The protein sequences of *A. ferrooxidans* (*Af*) (ATCC 23270 and ATCC 53993), *Bacillus subtilis* (*B. subtilis*), *Escherichia coli* (*E. coli*), *Enterococcus hirae* (*E. hirae*), and *Thermoplasma volcanium* (*T. volcaniu*) were used as references to search and compare the common conserved motif in the copper resistance proteins identified in the ACH strain (ACH) genome. As mentioned, four Cu-ATPase-like pumps were identified in the ACH genome (Figure 3). Specifically, one CopA protein with a high amino acid identity to CopA1_Af_ (98%) (AFE_2439; Lferr_2066) was previously identified in *A. ferrooxidans* [8,19]. In addition, three CopB proteins were identified; however, their amino acid identities were significantly lower with respect to CopB_Af_ protein (63%, 62%, and 62%, respectively) (AFE_2021; Lferr_1686). Moreover, P-ATPases transport several different compounds, including ions and phospholipids, across a membrane using ATP hydrolysis for energy. There are many different classes of P-ATPases, which transport specific types of ions: H^+^, Na^+^, K^+^, Mg^2+^, Ca^2+^, Ag^+^, Ag^2+^, Zn^2+^, Co^2+^, Pb^2+^, Ni^2+^, Cd^2+^, Cu^+^, and Cu^2+^ [54]. Nevertheless, the P-ATPases identified in *A. ferrivorans* ACH showed several of the characteristic conserved domains and motifs of copper transporter systems (Table 2). Furthermore, these heavy metal ATPases had been classified as CPx-type ATPases owing to the conserved motif (CPC/CPH/SPC) reported in most of these systems. It has been suggested that this domain yields information about the ion specificity for the protein [19].

In the particular case of copper, the most common motif is CPCALGLA. However, there are reports of motif change to CPHALGLA or CPCAMGLA in some CPx-type Cu-ATPase [55]. Therefore, CopA1*_ACH_* contained the same motif of ion specificity present in CopA1*_Af_*, CopA2*_Af_*, and CopB*_E.hirae_* reported previously (CPHALGLA). Moreover, the same 6’ translocation motif of CopB_Af_ was present in the three ACH-strain CopB proteins (CPCAMGLA) (CopB_ACH1_, CopB_ACH2_, and CopB_ACH3_) (Table 2). Additionally, the CPx conserved motif requirement for the proper Cu-ATPase function has been stated by some authors, who reported its active participation in metal binding. Then, the mutation of the CPH motif to SPH in CopB protein resulted in the loss of *Enterococcus hirae* copper resistance [56]. Similar results were observed when the Cys in the CPC motif of *Escherichia coli* CopA protein resulted in the loss of copper resistance [57].

**Figure 4:**
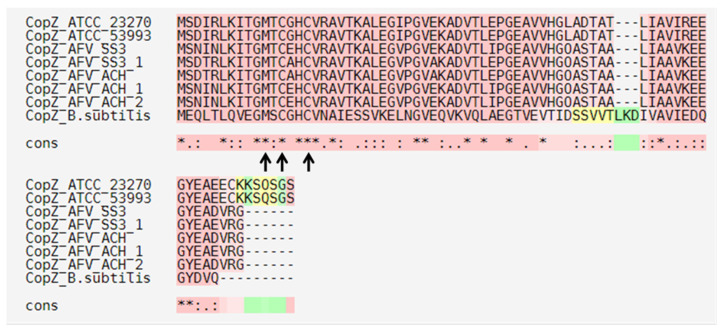
Amino acid sequence alignment of CopZ proteins identified in different copper-resistant microorganisms. Black arrows indicate the conserved MXCXXC motif that binds copper. ATCC_23270: *A. ferrooxidans* ATCC 23270; ATCC_53993: *A. ferrooxidans* 53,993; AFV_SS3: *A. ferrivorans* SS3; AFV_ACH: *A. ferrivorans* ACH; and B. subtilis: *Bacillus subtilis* 168.

On the other hand, we identified three metallochaperones in the ACH genome (Figure 2 and Figure 3) with high identity to *A. ferrooxidans* ATCC 23270 CopZ protein (AFE_1862) (82%, 82%, and 85%, respectively). This protein belongs to a family of highly conserved chaperones that have been suggested to transfer copper to the Cu-ATPases in yeast and bacteria [58,59]. These chaperones have a conserved metal-binding motif containing two important cysteine residues for metal ion binding and transfer (MXCXXC) [59]. Moreover, this motif has been identified in *B. subtilis* and *A. ferrooxidans*, among other microorganisms [59,60]. The amino acid sequence alignments of the three *A. ferrivorans* ACH-identified proteins (CopZ_AFV_ACH) against the *A. ferrooxidans* (ATCC 23270 and ATCC 53993), *A. ferrivorans SS3* (CopZ_AFV_SS3), and *B. subtilis* 168 orthologs showed that the conserved residues are, indeed, part of the copper-binding site (Figure 4).

Navarro and collaborators reported that the activity of *A. ferrooxidans* CopZ protein was lost when the conserved amino acids were mutated (Cys13Ser and Cys16Ser, copper-binding site); hence, the copper resistance capacity was lost when this was heterologously expressed in *E. coli* [60]. However, comparing the ACH CopZ proteins to those from *B. subtilis*, the amino acid identity percent decreases drastically (27%). As expected, ACH sequences are more related to those from Gram-negative microorganisms. Nonetheless, they all share the same copper-binding conserved motif. On the other hand, the genomic context of ACH CopZ proteins is organized as part of a gene cluster along with CopB (Figure 2). Contrarily, CopZ_Af_ does not form part of any gene cluster with other copper-related genes [60].

**Figure 5:**
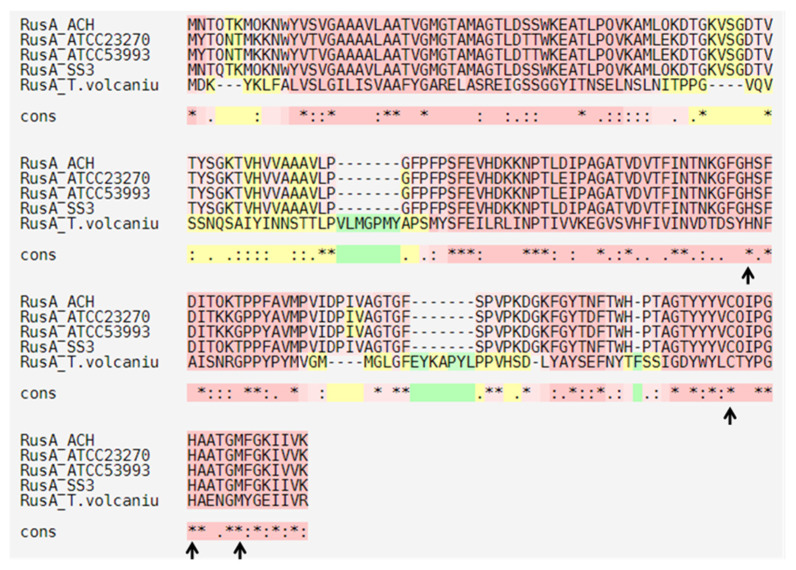
Amino acid sequence alignments of RusA proteins identified in different copper-resistant microorganisms. Black arrows indicate the conserved H-C-H-M motif that is part of the type I copper-binding of cupredoxin proteins. ATCC_23270: *A. ferrooxidans* ATCC 23270; ATCC_53993: *A. ferrooxidans* 53993; AFV_SS3: *A. ferrivorans* SS3; AFV_ACH: *A. ferrivorans* ACH; and T. volcaniu: *Thermoplasma volcanium*.

Using the same strategy, in the ACH genome, we identified two rusticyanin proteins (Figure 2), of which RusA has a high identity to the one previously reported for *A. ferrooxidans* RusA_Af_ (92%, AFE_3146). Rusticyanin proteins contain a mononuclear type I copper center, which is classified as a blue copper protein, owing to the intense blue color given by these centers (cupredoxins fold). In addition, the physiological role of most copper-binding proteins with cupredoxin folds is to mediate electron transfer or catalyze redox reactions [61]. Nevertheless, it has been proposed that cupredoxin-like proteins play the role of copper carriers to maintain copper homeostasis in some microorganisms [62,63]. Moreover, cupredoxin proteins have a characteristic conserved type I metal-binding motif (H-C-H-M) [62]. As seen in Figure 5, the amino acid sequence alignment of RusA_ACH_ against the ortholog from *A. ferrooxidans* (RusA_ATCC 23270 and RusA_ATCC53993), *A. ferrivorans* SS3 (RusA_SS3), and *Thermoplasma volcanium* (RusA_T. volcaniu) showed the conservation of the residues in the type I copper-binding motif (H-C-H-M). Interestingly, when one of these amino acids was mutated and heterologously expressed in *E. coli,* the protein lost its copper-binding ability and, consequently, its bacterial resistance capacity [60]. Hence, the protection granted by this protein has been attributed to its ability to bind copper, which is dependent on the H-C-H-M motif.

**Figure 6:**
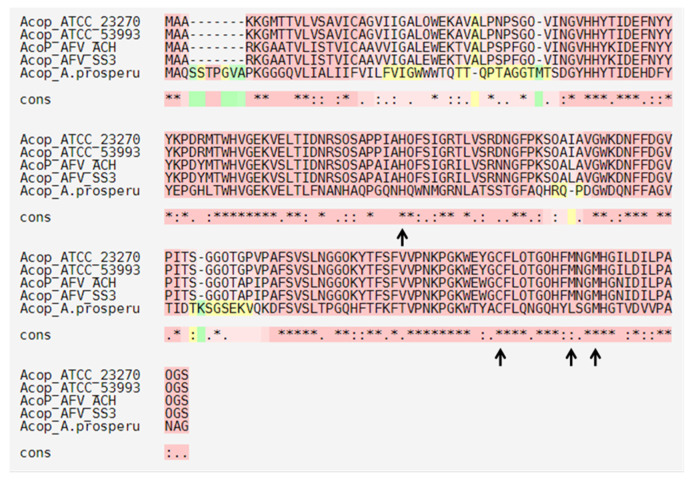
Amino acid sequence alignments of Acop proteins identified in different copper resistance microorganisms. Black arrows indicate the conserved H-C-H-M motif part of the type I copper-binding characteristic of cupredoxin proteins. ATCC_23270: *A. ferrooxidans* ATCC 23270; ATCC_53993: *A. ferrooxidans* 53993; AFV_SS3: *A. ferrivorans* SS3; AFV_ACH: *A. ferrivorans* ACH; and *A.prosperu*: *Acidihalobacter prosperus*.

The RusA protein identified in the *A. ferrivorans* ACH strain was found in a cluster organization along with several genes related to electron transport and energy production/conversion (Figure 2). Furthermore, Acop protein (“Acidophile cytochrome c partner“) is a cupredoxin that interacts with both cytochrome c and cytochrome c oxidase to maintain their optimal activity at physiological pH [64]. Hence, the Acop proteins have the same conserved type I metal-binding motif (H-C-H-M), identified previously in rusticyanin proteins (Figure 6). This conserved motif is essential for the correct functioning of Acop. Additionally, this was demonstrated in *A. ferrooxidans* ATCC 23270 Acop, mutating the amino acids involved in copper binding and through heterologous expression in a copper-hypersensitive *E. coli* strain [60].

Regarding RND systems, we identified five *cus* genes (Figure 2), six possible *cusCBA*-like clusters (18 genes), and their regulators *cusRS* (Figure S1) in the *A. ferrivorans* ACH genome. The Cus systems are normally composed of three proteins in an operon organization (*cusCBA*). CusA protein is an inner membrane transporter, which belongs to the resistance nodulation cell division family. It also works as a secondary transporter energized by proton-substrate antiport and is responsible for the substrate specificity. Meanwhile, the periplasmic adaptor factor protein CusB (family of membrane fusion protein, MFP) works as a link between CusA and the outer membrane factor (OMF) CusC [65,66]. Moreover, CusCBA systems, in some cases, have an additional component: the small periplasmatic copper chaperone CusF [67]. Additionally, CusF protein was identified in the ACH genome with a high identity percent (87%) regarding *A. ferrooxidans* ATCC 53993 (Lferr_0174) CusF3_Af_. Furthermore, this protein is located in the copper genomic island detected in the *A. ferroxidans* ATCC 53993, which is more resistant compared with *A. ferrooxidans* ATCC 23270, which does not have CusF [38]. Nevertheless, the *cusF3* gene in the ACH strain is not part of any cluster.

In addition, it has been described that the CusCFBA system regulation in *E. coli* would be under the CusSR two-component system control, which activates the expression in response to increased copper levels [68]. CusS protein encodes a cytoplasmic membrane histidine kinases sensor, probably sensing copper ions. Meanwhile, CusR protein encodes a response regulator (phosphate receiver) that activates the *cusCFBA* transcription [69]. Consequently, both CusRS proteins were identified in the ACH genome (Figure S1), sharing 37% and 55% amino acid identity with those characterized in the *E. coli* K-12 strain [68].

### 3.5. Effects of Copper in A. Ferrivorans ACH Gene Expression of Resistance Markers

**Figure 7:**
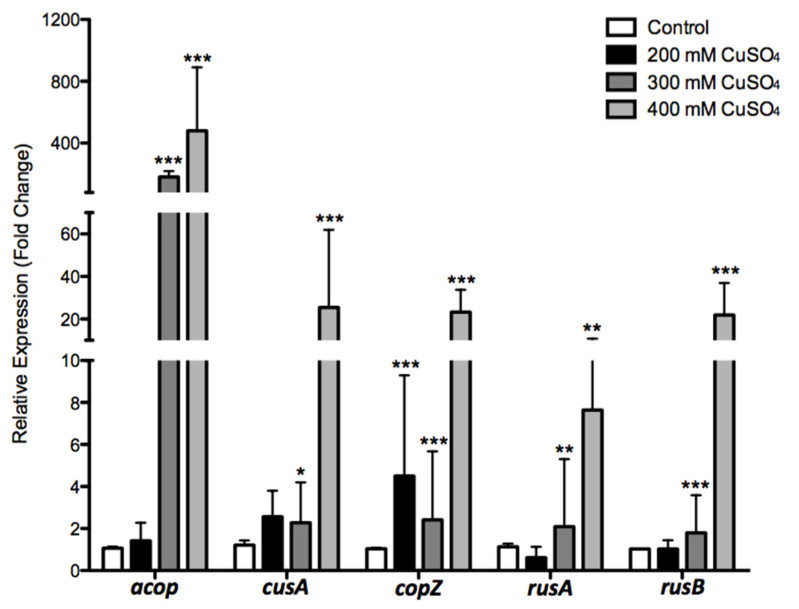
Gene relative expression of *A. ferrivorans* ACH selected genes, involved in copper resistance. The cells were grown in ferrous iron with the addition of three different concentrations of CuSO_4_ 200, 300, and 400 mM (grey to black bars are copper conditions and white bars are control conditions). Error bars indicate standard deviations based on three different experimental values. Applications of Student’s *t*-test were as follows: *** *p* ≤ 0.001, ** *p* ≤ 0.01, and * *p* ≤ 0.05.

To cover all the previously discussed systems, five representative genes (*cusA3*, *copZ*, *rusA*, *rusB,* and *acop*) were selected for expression experiments [18,41,58,60,62]. For these assays, the ACH strain was grown in the presence of 0, 200, 300, and 400 mM of CuSO_4_, which were selected as the experimental conditions. As seen in Figure 7, expression analysis showed that, against 200 mM of CuSO_4_, three genes increased expression levels: *acop*_ACH_ (1.4-fold), *cusA*_ACH_ (2.5-fold), and *copZ*_ACH_ (4.5-fold), which was the only significative. On the other hand, *rusA*_ACH_ showed no significative decrease, and no change was observed in the *rusB*_ACH_ expression level. Furthermore, at 300 mM CuSO_4_, we observed a significant up-regulation in all five analyzed genes. There was a 180-fold up-regulated expression of *acop*_ACH,_ followed by *copZ*_ACH_ (2.4-fold) and *cusA3*_ACH_ (2.2-fold). Conversely, both *rusA*_ACH_ and *rusB*_ACH_ genes slightly increased their expression levels similarly under these conditions (2-fold and 1.7-fold, respectively).

In the presence of 400 mM of CuSO_4_, a significant increase in the expression levels of all five genes was observed, greater as compared with the two previous conditions. The *acop* cupredoxin again showed the higher increase (about 480-fold), followed by the inner membrane transporter (*cusA*_ACH_ gene), which reached 25-fold. Interestingly, both rusticyanins increased their expression levels by about 22-fold and 7.6-fold (*rusB*_ACH_ and *rusA*_ACH_, respectively). Finally, CopZ chaperone increased its expression by about 23-fold. All these results suggest that all measured genes could play an important role in the copper resistance of *A. ferrivorans* ACH, and the up-regulation follows a proportional pattern regarding copper concentration, which is common for adaptive responses.

The activation of most genes in the copper repertoire described for *Acidithiobacillus* has been reported in response to different copper concentrations. This was the case for the ATCC 23270 strain grown with 25 mM of CuSO_4_ [19]. Furthermore, similar results were obtained at proteomic level when the strain ATCC 23270 was grown with 40 mM of CuSO_4_, increasing the expression of CusA, CusB, and CusC [41]. On the other hand, the *A. ferrooxidans* ATCC 53993 strain showed a higher copper resistance level (>100 mM CuSO4) regarding the ATCC 23270 strain, which is related to a 160 kb genomic island, with additional copper resistance genes [38]. Those additional genes include two RND systems (*cusCBA*2 and *cusCBA*3), two chaperones (*cusF*3 and *cusF*4), and one P-type ATPase (*copA*3), which were overexpressed in response to the 40 mM of CuSO4 [70]. Finally, the participation of two iron oxidation proteins (Rus and Acop) has been suggested in *Acidithiobacillus* copper resistance [41,60,71].

The periplasmic Acop protein from *A. ferrooxidans* was characterized for the first time by Roger and collaborators. This has a type I copper-binding site (green site) different from the other cupredoxin families like rusticyanins (blue site). Moreover, this protein is involved in the respiratory pathways of acidophilic microorganisms, as part of the *rus* operon [71]. Additionally, the increased transcriptional level of this gene has been reported previously for *A. ferrooxidans* in response to copper. Particularly, Felicio and collaborators described for *A. ferrooxidans LR* the induction of a 17 kDa protein in the presence of 200 mM of copper, suggesting that it was probably a rusticyanin [72]. In addition, Almárcegui and collaborators reported a 2.9-fold transcriptional increase of rusticyanin from ATCC 23270 (AFE_3151) when this strain was grown in sulfur and 50 mM of CuSO_4_ [41]. Moreover, Navarro and collaborators reported a fivefold transcriptional level increase of *acop* when *A. ferrooxidans* ATCC 23270 was exposed to 40 mM of CuSO_4_ and ferrous sulfate as an energy source [60]. Additionally, Martinez-Bussenius and collaborators reported, for *A. ferrooxidans* ATCC 53993 *acop* (Lferr_2749), a 1.55-fold up-regulation under the same condition [70].

Interestingly, it has been suggested that Acop protein could have two roles in *A. ferrooxidans*: (i) acting chaperone-like and (ii) acting as a link between cytochrome c and cytochrome c oxidase [64]. Additionally, Zhang and collaborators suggested that the Acop protein could act like the periplasmic copper chaperone CopC (carrying copper for CopA and/or CopB proteins), identified in the *Pseudomonas syringae cop* operon [73]. Moreover, CopC protein, as well as Acop, have a cupredoxin fold, which could imply similar functions in the ACH strain copper resistance. All these works support our findings and strongly suggest the important role that Acop protein could play in the resistance that acidophilic microorganisms present to high copper concentrations.

On the other hand, *copZ*_ACH_ was the second-highest up-regulated gene in the 400 mM of CuSO_4_ condition. While this cytoplasmic chaperone transports copper to a Cu-ATPase in *B. subtilis* and *E. hirae* [74,75], its role for acidophilic microorganisms was unclear. Hence, Navarro and collaborators reported, for the first time, CopZ’s participation in *A. ferrooxidans* ATCC 23270 copper resistance. Furthermore, the expression of *copZ_Af_* (AFE_1862) was up-regulated (fourfold) in the presence of 20 mM of CuSO_4_. Then, when the cells were grown in 40 mM of CuSO_4_, the transcriptional level was up-regulated (threefold) at a lower magnitude [60]. In the particular case of the ACH strain, the presence of three *copZ* copies and the up-regulation when cells were grown at high copper concentrations allow us to suggest that this protein would actively participate in copper resistance as in *B. subtilis*. Thus, helping to reduce the amount of free copper in the cytoplasm, together with an ATPase, contributes to copper detoxification.

Regarding the transcription of *cusA*, it was the third gene with the highest expression level change in the 400 mM of CuSO_4_ condition. In addition, Orellana and collaborators reported the existence of a 160 kb genomic island, exclusive to the ATCC 53993 genome, which contains a *cusA3* gene [38]. This genomic island conferred a greater copper resistance to ATCC 53993 as compared with ATCC 23270 (which does not have this island). Moreover, the up-regulation of *cusA3* for ATCC 53993 was reported in response to 40 mM of CuSO_4_ [70]. The expression level for *cusA* in the ACH was 10 times higher compared with the ATCC 53993 strain. We must consider that both copper conditions were different and that the response is related to the strain resistance level and could also be proportional to the CuSO_4_ concentration. Furthermore, the strong *cusA* induction in response to copper was described for other organisms, such as *E. coli* and *Shewanella* [67,76]. Hence, the presence of this gene can be considered a competitive advantage over other microorganisms.

**Figure 8:**
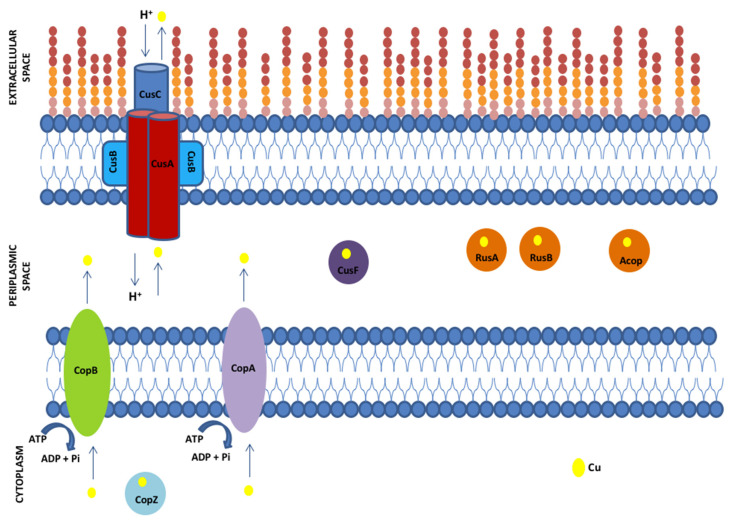
Working scheme showing the identified proteins with potential participation in copper resistance in *A. ferrivorans* ACH. Proteins: CopZ (metallochaperone), CopA (P-type ATPase pump), CopB (P-type ATPase pump), RusA (rusticyanin A), RusB (rusticyanin B), Acop (cupredoxin), CusF (periplasmic metallochaperone), CusA (inner membrane transporter (RND family)), CusB (periplasmic adaptor factor protein (MFP family)), and CusC (outer membrane factor (OMF)). ATP (adenosine triphosphate), ADP (adenosine diphosphate).

Interestingly, the transcriptional levels of rusticyanin genes (*rusA* and *rusB*) from the ACH strain increased under higher copper conditions. Similar results were reported for *A. ferrooxidans* ATCC 23270 *rusA* (AFE_3146) when grown in the presence of 20 mM of CuSO_4_, increasing the transcriptional levels by fourfold [60]. Particularly, the *rusB* gene is absent from several *Acidithiobacillus* members, which were up-regulated in the ACH strain in response to copper. Importantly, when the Acop, Rus, and CopZ proteins from *A. ferrooxidans* ATCC 23270 were heterologously expressed in a copper-sensitive *E. coli*, all of them conferred resistance to copper [60]. The presence of rusticyanins and other advantageous genes in the ACH strain could be considered a great benefit regarding most of the microorganisms that are frequently used in the industry, which promotes the use of this strain for higher copper concentration processes. A summary model illustrating the products of the main genes identified in the *A. ferrivorans* ACH genome potentially involved in high copper resistance is presented in Figure 8.

In aerobic systems, the Cu^2+^ that is supplemented in the culture media is reduced to Cu^+^ once it encounters the microorganism’s respiratory chain. As has been mentioned previously, the Cu^+^ is the most toxic form of this metal. As copper enters freely into the cell (because this is essential for several proteins and a cofactor for some enzymes), it can be accumulated in the cytoplasm, becoming toxic. Thus, bacteria in these environments must have machinery to cope with this, avoiding the harmful effects. Specifically, the ACH strain is able to expulse the copper out of the cell. To begin, copper is captured by the CopZ chaperone that carries the metal ion toward the P-ATPases (Cop proteins), which, in turn, pumps it toward the periplasmic space. Then, once the metallic ions are in the periplasmic space, the CusF chaperone binds to the copper and carries it to the RND system (Cus proteins, which go through the membrane), which expels it to the exterior. On the other hand, when the microorganism is exposed to very high concentrations of copper, both Rus and Acop proteins act as chaperones that sequester the copper, diminishing the toxic effects of this metal and protecting the electron chain, while the active machinery (Cop and Cus systems) expels the excess from the cell to avoid damages. The extra copies of *copB* and *copZ* genes could be the determining factor generating the ACH strain high copper resistance level, as it would expel Cu to the periplasm with greater avidity compared with the SS3, CF27, YL15, and PQ33 strains.

In addition to the previously mentioned advantages, compared with the reports on copper leaching at mesophilic temperatures [77], the use of psychrotolerant bacteria avoids the generation of some iron precipitates (like jarosite) that accumulate on the mineral surfaces. Furthermore, it has been confirmed that the formation of these problematic precipitates is much lower or does not occur at low temperatures [78]. Hence, the bioleaching at low temperatures would be advantageous, so it becomes necessary to identify and use psychrotolerant leaching organisms tolerant to copper like *A. ferrivorans*, in order to recover interest metals.

## 4. Conclusions

We identified at least fifteen genes potentially involved in copper resistance in the *A. ferrivorans* ACH strain, namely, several Cu-ATPase pumps, the RND system, metallochaperones, Rusticyanin, and the periplasmic Acop protein. The ACH genome has several copies of some of these genes that could respond when the growth conditions become unfavorable or in the presence of increasing concentrations of copper, implying a competitive advantage. Our expression results suggest that, in the presence of high copper concentrations, the ACH strain activates several genes of its copper responsive repertoire (*acop, rus, copZ,* and *cusA*), which are suggested to play an important role in copper resistance in the common bioleaching microorganism. This work represents the first report of active mechanisms of copper resistance for the *A. ferrivorans* species and, more importantly, the ability to resist the Cu at low temperatures. Hence, the combination of these two mechanisms could bring application advantages, reducing industrial costs. Nonetheless, clarifying the physiological differences between the copper leaching carried out by thermophilic, mesophilic, and psychrophilic microorganisms should be an important focus for future investigations. On the other hand, elucidating other determinants that could contribute to the global resistance to high copper concentrations remains a pending goal, considering functional approaches such as proteomics and molecular recombination studies, as well as to shed light on the participation of the *cusCBA-*like genes, which are many and are found in all of the members of the *A. ferrivorans* species.

## Figures and Tables

**Table 1:** Interest copper resistance markers from *Acidithiobacillus.* MFP, membrane fusion protein.

Protein	Functional Description *
**CopA**	Copper-exporting P-type ATPase A, exports Cu^+^ from the cytoplasm to the periplasm; binds 2 Cu^+^ ions per monomer, which are transferred to periplasmic copper chaperone CusF upon ATP hydrolysis.
**CopB**	Copper-exporting P-type ATPase B, copper-translocating outer membrane protein.
**CopZ**	Copper chaperone, ion-binding protein delivering two Cu^+^ ions to the copper-transporting ATPase CopA.
**CusA**	Cation efflux system protein, copper efflux RND transporter permease.
**CusB**	Cation efflux system protein, copper efflux pump MFP component.
**CusC**	Cation efflux system protein RND transporter outer membrane channel component
**CusF**	Cation efflux system protein, periplasmic copper-binding chaperone component.
**RusA**	Rusticyanin type A, high potential iron sulfur protein, blue copper cupredoxin.
**RusB**	Rusticyanin type B, high potential iron sulfur protein, blue copper cupredoxin.
**Acop**	Acidophile cytochrome c oxidase partner, green copper cupredoxin [5].

* information collected from UniProt (https://www.uniprot.org/).

**Table 2:** Conserved motif identified in the CPx-type ATPases found in the *A. ferrivorans* ACH genome and other microorganisms with similar copper resistance systems. Af: *Acidithiobacillus ferrooxidans*; ACH: *Acidithiobacillus ferrivorans* ACH (Modified from [19]).

Protein	Metal Binding Motif	Phosphatase Domain	6′ Translocation Motif	Phosphorylation Domain	Conserved GXGXXG/A Motif	TGDN Motif	GDGXNDXP Motif
CopA (*E.coli*)	CASC….CASC	TGEP	CPCALGLA	FDKTGTLT	GLGVSG	TGDN	GDGINAP
CopA (*E.hirae*)	CANC	TGES	CPCALGLA	LDKTGTLT	GAGISG	TGDN	GDGINAP
CopB (*E.hirae*)	No	TGES	CPHALGLA	LDKTGTLT	GVGLEA	TGDN	GDGINDAP
CopA1_Af_	No	TGES	CPHALGLA	FDKTGTLT	GKGAQA	TGDS	GDGVNDAP
CopA2_Af_	No	TGES	CPHALGLA	FDKTGTLT	GKGAQA	TGDS	GDGVNDAP
CopB_Af_	CASC….CASC	TGEP	CPCAMGLA	LDKTGTLT	GKGVRG	TGDL	GEGINDSP
CopA1_ACH_	No	TGES	CPHALGLA	FDKTGTLT	GKGAQA	TGDS	GDGVNDAP
CopB_ACH1_	CASC….CASC	TGEP	CPCAMGLA	FDKTGTLT	GYGVEG	TGDA	GDGINDAP
CopB_ACH2_	CASC….CASC	TGEP	CPCAMGLA	FDKTGTLT	GYGIEG	TGDG	GDGINDAP
CopB_ACH2_	CASC….CASC	TGEP	CPCAMGLA	FDKTGTLT	GYGIEG	TGDG	GDGINDAP

## Supplementary Materials

The following are available online at https://www.mdpi.com/2073-4425/11/8/844/s1, Figure S1: Organization of the *cusCBA*-like gene involved (potentially) in copper resistance and found in the *A. ferrivorans* ACH genome. *cusA-like*: inner membrane transporter (RND family); *cus-like:* periplasmic adaptador factor protein (MFP family); *cusC-like:* outer membrane factor (OMF); *ompR:* transcriptional regulator protein; *ppK*: polyphosphate kinase; *foldD*: bifunctional protein; *cusR:* phosphate receiver response regulators; *cusS:* sensor histidine kinases; Figure S2: Genetic organization and similarity of some relevant copper resistance genes found in *A. ferrivorans*. Heat scale shows sequence identity percentage of all strains’ genomes available in databases using the *A. ferrivorans* SS3 genes as references. *cusC*: outer membrane factor (OMF); *cusB*: periplasmic adaptor factor protein (MFP family); *cusA*: inner membrane transporter (RND family); cb561: cytochrome b561; *cusF*: periplasmic metallochaperone; Figure S3: Additional genetic organization and similarity of potential copper resistance genes identified in *A. ferrivorans*. Heat scale shows sequence identity percentage of all strains’ genomes available in databases using the *A. ferrivorans* SS3 genes as reference. *copB*: ATPase P-type; HP: hypothetical protein; *copZ*: metallochaperone; *rusA*: rusticyanin A; *Cco III*: cytochrome c oxidase polipeptide III; *CcoI*: cytochrome c oxidase polipetide I; *Cco II*: cytochrome c oxidase polypeptide II; *acop*: cupredoxin; *pilT*: virulence protein; *cusF*: periplasmic metallochaperone; *rusB*: rusticyanin B; *glx*: glutaredoxin; Table S1: Sequences used as a reference for the interest markers for comparative analyses; Table S2: Sequences of the identified interest markers extracted from the *A. ferrivorans* ACH genome; Table S3: Reference *A. ferrivorans* genomes available on the GenBank and used for the interest markers searches; Table S4: Oligonucleotide primers used for the gene relative expression experiments; Table S5: Potential genes involved in copper resistance (*cusCBA*-like) in all strain of the *A. ferrivorans* species. Sequence identity relative to the SS3 strain.
