# Supplementary material for: Determinants of Copper Resistance in Acidithiobacillus Ferrivorans ACH Isolated from the Chilean Altiplano
Source: Genes (Basel). 2020 Jul 24;11(8):844. doi: 10.3390/genes11080844 (PMC7463520; doi:10.3390/genes11080844)
Supplement: Supplementary file 1 [file genes-11-00844-s001.pdf]

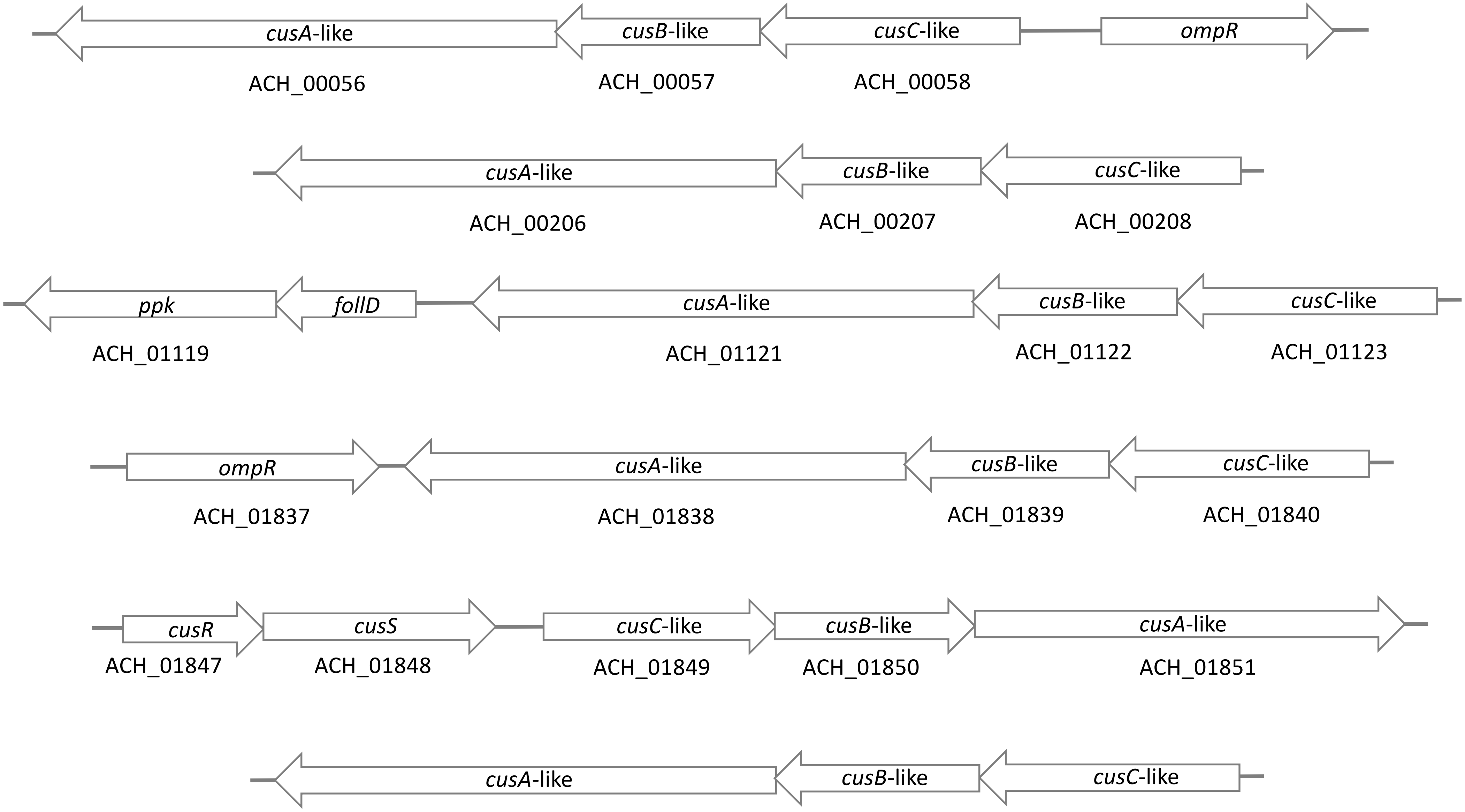

Figure S1. Organization of the *cusCBA*-like gene involved (potentially) in copper resistance and found in the *A. ferrivorans* ACH genome. ***cusA-like:*** inner membrane transporter (RND family); ***cus-like:*** periplasmic adaptador factor protein (MFP family); ***cusC-like:*** outer membrane factor (OMF); ***ompR:*** transcriptional regulator protein**; *ppK*:** polyphosphate kinase**; *foldD*:** bifunctional protein; ***cusR:*** phosphate receiver response regulators; ***cusS:*** sensor histidine kinases.

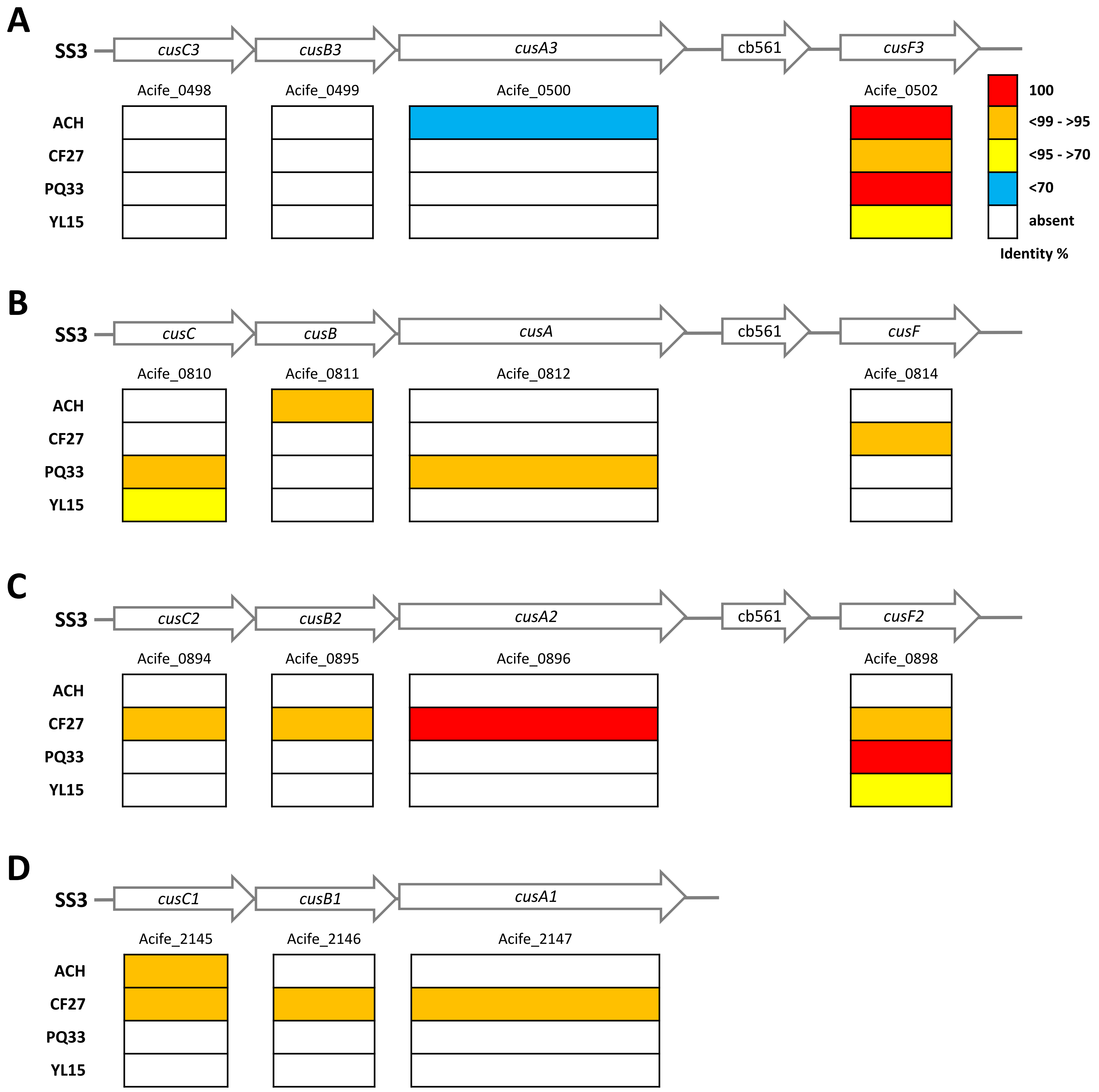

Figure S2. Genetic organization and similarity of some relevant copper resistance genes found in *A. ferrivorans*. Heat scale shows sequence identity percentage of all strains' genomes available in databases using the *A. ferrivorans* SS3 genes as references. ***cusC*****:** outer membrane factor (OMF)**;** ***cusB***: periplasmic adaptor factor protein (MFP family); ***cusA*****:** inner membrane transporter (RND family); **cb561**: cytochrome b561; ***cusF***: periplasmic metallochaperone.

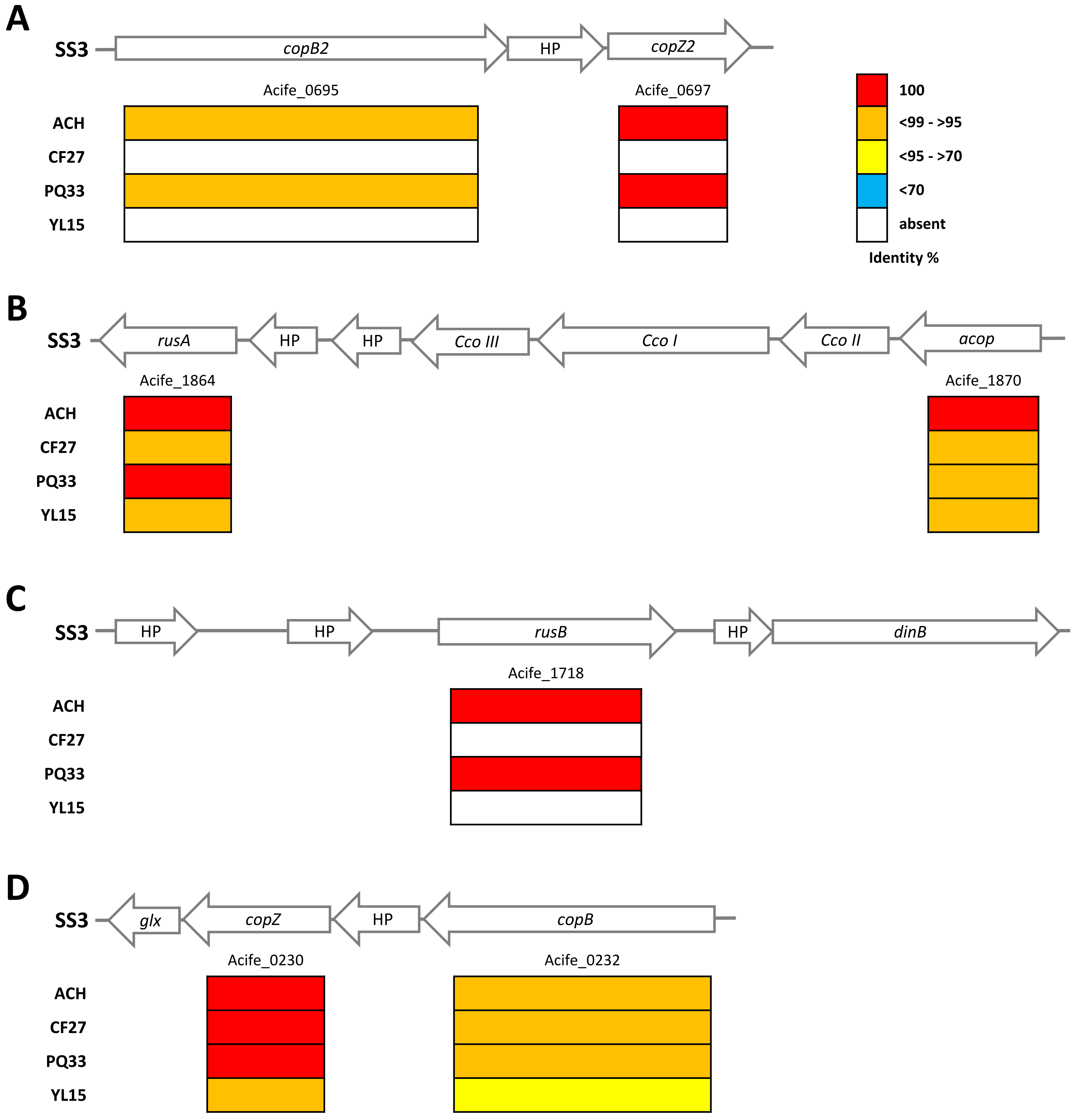

Figure S3. Additional genetic organization and similarity of potential copper resistance genes identified in *A. ferrivorans*. Heat scale shows sequence identity percentage of all strains' genomes available in databases using the *A. ferrivorans* SS3 genes as reference. ***copB***: ATPase P-type; **HP**: hypothetical protein; ***copZ***: metallochaperone; ***rusA***: rusticyanin A**;** ***Cco III***: cytochrome c oxidase polipeptide III; ***CcoI***: cytochrome c oxidase polipetide I; ***Cco II***: cytochrome c oxidase polypeptide II; ***acop***: cupredoxin; ***pilT*****:** virulence protein **;** ***cusF***: periplasmic metallochaperone; ***rusB***: rusticyanin B; ***glx***: glutaredoxin.

Table S1. Sequences used as a reference for the interest markers for comparative analyses.

Proteins	Organisms	UNIPROT Accession
CopA (Afe_2439)	*A. ferrooxidans* ATCC 23270	B7J6X9
CopB (Afe_2021)	*A. ferrooxidans* ATCC 23270	B7J4N2
CopB (EHR_09090)	*E. hirae* ATCC 9790	P05425
CopZ (Afe_1862)	*A. ferrooxidans* ATCC 23270	B7JBW5
CopZ (BSU33510)	*B. subtilis* 168	O32221
CopZ (EHR_09080)	*E. hirae* ATCC 9790	Q47840
RusA (Afe_3146)	*A. ferrooxidans* ATCC 23270	B7JAQ0
RusA (TVG1528192)	*T. volcanium* GSS1	Q978E0
Acop (Afe_3151)	A. ferrooxidans ATCC 23270	B7JAQ5
Acop (N/A)	*A. prosperus* V6	B2ZFM9
CusF3 (Lferr_0174)	*A. ferrooxidans* ATCC 53993	*IPR021647
CusR (CUSR_ECOLI)	*E. Coli* K-12	P0ACZ8
CusS (CUSS_ECOLI)	*E. Coli* K-12	P77485

* InterPro accession

Table S2. Sequences of the identified interest markers extracted from the *A. ferrivorans* ACH genome.

Protein	Sequence
HF929_13905 CopZ	MSDTRLKITGMTCAHCVRAVTKALEGVPGVAKADVTLEPGEAVVHGQASTAALIAAVKEEGYEAEVRG
HF929_13895 CopB	MEDEKRGHLEIGIEGMTCASCSARVERALGKLPGVTSASVNLATERAEVLFDPQQLDAARIAEAIQATGYAPVTDEIDLAVEGMTCASCVGRVERALRQQPGVLEAAVNLAT<br>ERALVRYIPAMVGMDELATAVSAAGYAAHPVQENGEQDTDQRRASLRAMGRDVIVAVALATVILVLAMGTAFVPALHRALAAASPFAHFWEWVQFVLASIVLFGPGRRF<br>FRPGLIAYRHLSPDMNSLVATGTGAAWAYSVLVLLVPALFPAEAQHVYFDSAAVVIAAVLAGKYLEALAKGRTSSAIRKLVGLQAKTAHRLDENGAEHDVPVSRLRTGERIVI<br>RPGERIPVDGRVLEGRAHVDEAMLTGEPIPSAKDLNDAVVGGTVCQDGRLVVEATSVGRDTVLAQIIHLVESAQTGKLPIQGLTDRAVRVFTPVVLVIALATFGVWVALTGN<br>VSVALVTAVAVLVVACPCAMGLATPAAIMVGTGRAAELGVLFRRGEALETLSHVDTILFDKTGTLTEGHPALVDQGGGAAETALRLAAALESASEHPLGRAIVAAAGERGM<br>NIPAVKDFRAIAGYGVEGEVEGRMVRVGARRFMERENVALGDDAEIAARLEGTGRTVVFVAVDATLLGWLAIADRIKPEARAVVQALRERGLQVAMVTGDARATAQSVA<br>TELRIEEMHAEVLPEDKAKVVTGLQEQGRRVAFVGDGINDAPALAQADVGIALASGTDIAIEAADVTLTRGQLGEVVTALNAARRTLGNIRGNLFWAFFYNILLIPIAAGVAA<br>PIGIHLNPMVAGVAMGLSSVFVLGNSLRLKRLKAYIPLITPGAVKHTALTPAHS
HF929_07940 CopZ2	MSNINLKITGMTCEHCVRAVTKALEGVPGVEKADVTLIPGEAVVHGQASTAALIAAVKEEGYEADVRG
HF929_07930 CopB2	MDDKTRGHLEIGIDGMTCASCSARVERALGKLPGVTSASVNLATERAEVFFDPQQLDAARIAEAIRETGYTPVTDEIDLVVEGMTCASCVGRVERALRQQSGVLEAVVNLAT<br>ERARVRYIPAMISTDELASAVSAAGYAAHPVQEDGEQDVNEADQRRVSLRAMGRDVIVAAVLAMAILVLSMGASFVPAFDHALMVASPFAHFWEWVQFVLASIVLFGPG<br>RRFFRPGLIAYRHLSPDMNSLVATGTGAAWAYSVLVLLAPVLFPPEAQHVYFDSAAVVIAAVLAGKYLEGLAKGRTSSAIRKLAGLQAKTAHRLDANGIEQEVPVSRLRTGER<br>VVVRPGERIPVDGRVVEGRAHVDEAMLTGEPLPSAKTMDDTVVGGTICQDGRLVVEATSVGRDTVLAHIIHLVESAQTGKLPIQGLTDRAVRVFTPVVLVIALTTFGVWVAL<br>TGNVSIALVAAVAVLVVACPCAMGLATPAAIMVGTGRAAELGVLFRKGEALETLSHVDALLFDKTGTLTEGHPALVDQGGPDPATALRLAAALESASEHPLGRAIVTAAGER<br>GMHLPTVKNFRSIAGYGIEGEVQGRLVHVGARRFMERENVLLGDNAETAAQRENDGRTVVFVAVDATLIGWLAIADRIKPEAYAVVQALRVRGLQVAMVTGDGRATAQS<br>VARQLHIEQVHAEVLPQDKAKVVTQLQEQGRRVAFVGDGINDAPALAQANVGIALASGTDIAIEAADVTLTRGQLGEVVTALTAARRTLSNIRGNLFWAFFYNILLIPVAAG<br>VAVPIGIHLNPMVAGVAMGLSSVFVLSNSLRLKRLKAYVPTVTPGAVQNAALEPAHS
HF929_07940 CopZ3	MSNINLKITGMTCEHCVRAVTKALEGVPGVEKADVTLIPGEAVVHGQASTAALIAAVKEEGYEADVRG
HF929_07930 CopB3	MDDKTRGHLEIGIDGMTCASCSARVERALGKLPGVTSASVNLATERAEVFFDPQQLDAARIAEAIRETGYTPVTDEIDLVVEGMTCASCVGRVERALRQQSGVLEAVVNLAT<br>ERARVRYIPAMISTDELASAVSAAGYAAHPVQEDGEQDVNEADQRRVSLRAMGRDVIVAAVLAMAILVLSMGASFVPAFDHALMVASPFAHFWEWVQFVLASIVLFGPG<br>RRFFRPGLIAYRHLSPDMNSLVATGTGAAWAYSVLVLLAPVLFPPEAQHVYFDSAAVVIAAVLAGKYLEGLAKGRTSSAIRKLAGLQAKTAHRLDANGIEQEVPVSRLRTGER<br>VVVRPGERIPVDGRVVEGRAHVDEAMLTGEPLPSAKTMDDTVVGGTICQDGRLVVEATSVGRDTVLAHIIHLVESAQTGKLPIQGLTDRAVRVFTPVVLVIALTTFGVWVAL<br>TGNVSIALVAAVAVLVVACPCAMGLATPAAIMVGTGRAAELGVLFRKGEALETLSHVDALLFDKTGTLTEGHPALVDQGGPDPATALRLAAALESASEHPLGRAIVTAAGER<br>GMHLPTVKNFRSIAGYGIEGEVQGRLVHVGARRFMERENVLLGDNAETAAQRENDGRTVVFVAVDATLIGWLAIADRIKPEAYAVVQALRVRGLQVAMVTGDGRATAQS<br>VARQLHIEQVHAEVLPQDKAKVVTQLQEQGRRVAFVGDGINDAPALAQANVGIALASGTDIAIEAADVTLTRGQLGEVVTALTAARRTLSNIRGNLFWAFFYNILLIPVAAG<br>VAVPIGIHLNPMVAGVAMGLSSVFVLSNSLRLKRLKAYVPTVTPGAVQNAALEPAHS
HF929_12535 CopA	MAYAKAKDIGFEPPKEFKAIPGKGAQAVVNGRNVKVVSPGYLKEHGLDVSDERIAALASQGKTVIYLLVDEKPAGAVALADIIRAESREALARLKGMGVQVMMLTGDSEAV<br>ARWVAQEMGLDDYFAEVLPGQKAEKIKEVKARGLRVAMVGDGVNDAPALVEADVGIAIGAGTDVAIESADIVLVRSDPRDVVAILELSRATYRKMIQNLWWGAGYNIVAI<br>PLAAGVAYSVGLVLSPAVGAALMSVSTVIVAINAKLLQRVRLTA
HF929_14335 CusA	MTXTLVVGGLLPIMFSHGAGADVMKRXAAPMVGGMFSAALLALLVIPALYALWQKRRLSLR
HF929_14620 CusB	MRLHLPAYPGKSWEGRLNFLYPTLDPKNRTVTARLSFPNPGGTLRPGTYADATVLASPEETLAVPSSAVLRTTQGDYVMLGEGQGHFLPVQVALGPEADGWVAIDKGLKA<br>GDRVVESAQFLLYSESQFQSVKARMLGGTTSAAPGTGISQPQNMTQGRKPPASAAPAGNAGAPPTPPASSGPGVMVGMNRGQGGKSHD
HF929_13810 CusF	MVNVAMGPVKALGWPSMSMNFLLQNKAMLNGYKAGEMVKFSFAKDAAGGYIITRITPVRQ
HF929_14325 CusC	MYANIPGRFIQPTWGSIRLSVGVLLLLSATSAGAASLSLRDAEAIALRQNPGLGALTQKIAELRHKAVAVAQLPDPHLDLGALNLPLNSFSMNQQQMSMLSVGLSQTFPSFG<br>KLGLEGQQAGIEAQAAADTLRGQSAELVLLLRRAWLQALYTENAMATVRHQEQLEAESVQAALALYRSAQGSQAEVLRAQLARDNLANDISKLQAERASDLAQIAQILNLP<br>EPPSIEKQWPNLPPPPTLAEMEARLSGQPLLRAAQAQTRAAQMGVQVAKTGYWPDVTVSVGYGQDFYPGSPNWLSAGVNLSLPIFPGDRQDQDVAAAQARAQQAQYR<br>YDDQHLALTQQARAAFARYESYKIQLQRMDRQLLPTARNAFSATLAAYSAGRAGLNTVLRTQKEVLDYALTRLQYRRDLAISAAELDFLTTQGEMQP
HF929_15330 CusC2	MLLRRAWLQALYTENAMATVRHQEQLEAESVQAALALYRSAQGSQAEVLRAQLARDSLANDITRLQAEQASDLAQIAQILNLPEPPSIEKQWPNLPPPPTLAEMEARLSGQ<br>PLLRAAQAQTRAAQMGVQVAKTGYWPDVTVSVGYGQDFYPGSPNWLSAGVNLSLPIFPGNRQDQDVAAAQARAQQAQYRYDDQHLALTQQARAAFARYESYKIQLQR<br>MDRQLLPTARNAFSATLAAYSAGRAGLNTVLRTQKEVLDYALTRLQYRRDLAISAAELDFLTAQGEMQP
HF929_11640 RusA	MNTQTKMQKNWYVSVGAAAVLAATVGMGTAMAGTLDSSWKEATLPQVKAMLQKDTGKVSGDTVTYSGKTVHVVAAAVLPGFPFPSFEVHDKKNPTLDIPAGATVDV<br>TFINTNKGFGHSFDITQKTPPFAVMPVIDPIVAGTGFSPVPKDGKFGYTNFTWHPTAGTYYYVCQIPGHAATGMFGKIIVK
HF929_15015 RusB	MNTQIKPTMHKGRYIAAGLSTVLAAALGMSTAMAAPLDTSWKMATLPQVKALLAKDSGTVSGKTVTYSGKTVHVVAAAVLPGFPFPSFEIHDVKNPTLEIPAGATVDITFIN<br>TNKGFGHSLDITKKGPPYAVMPAINPIIAGTGFSPVPKSGGFGYTDFTWHPTAGTYYYVCQIPGHAATGMFGKIVVK
HF929_11610 Acop	MAARKGAATVLISTVICAAVVIGALEWEKTVALPSPFGQVINGVHHYKIDEFNYYYKPDYMTWHVGEKVSLTIDNRSQSAPAIAHQFSIGRILVSRNNGFPKSQALAVGWKD<br>NFFDGVPITSGGQTAPIPAFSVSLNGGQKYTFSFVVPNKPGKWEWGCFLQTGQHFMNGMHGNIDILPAQGS

Table S3. Reference *A. ferrivorans* genomes available on the GenBank and used for the interest markers searches.

Strain		Origin	BioProject	BioSample	Assembly Accession
A. ferrivorans	**PQ33**	Perú	SAMN04613415	PRJNA317458	GCA_001857665.2
A. ferrivorans	**YL15**	China	SAMN05353865	PRJNA327612	GCA_001685225.1
A. ferrivorans	**SS3**	Russia	SAMN00713557	PRJNA61509	GCA_000214095.3
A. ferrivorans	**CF27**	USA	SAMEA3139097	PRJEB5721	GCA_000750615.1

Table S4. Oligonucleotide primers used for the gene relative expression experiments.

Primer	Oligonucleotide sequence
16S Fw	5´ CGGCATCAGTCTGGAAGAG 3´
16S Rv	5´ TGGTCTGGATGGCAATAACA 3´
cusA Fw	5´ GACGGGTGGATATGCTGACT 3´
cusA Rv	5´ GGTGTGGATGACGATGTAGC 3´
copZ Fw	5´ GCATGACCTGCGAGCATT 3´
copZ Rv	5´ ATCCGCCTCATAGCCTTCTT 3´
acop Fw	5´ GGCTACGGTGCTTATCTCCA 3´
acop Rv	5´ GCGGGTGCTGACTGTGA 3´
rusA Fw	5´ TGACCTTCATCAACACCAACA 3´
rusA Rv	5´ GGATGCCAAGTGAAGTTCGT 3´
rusB Fw	5´ TCACCTTTATCAACACCAACAA 3´
rusB Rv	5´ CCAAGTGAAGTCCGTGTATCC 3´

Table S5. Potential genes involved in copper resistance (*cusCBA*-like) in all strain of the *A. ferrivorans* species. Sequence identity relative to the SS3 strain.

Gene	AFV SS3	ACH	CF27	PQ33	YL15
cusA-like	Acife_0050	ACH_00056 (100%)	CF27_01466 (96,98%)	PQ33_01064 (99,5%)	YL15_ 00559 (96,98%)
cusB-like	Acife_0051	ACH_00057 (100%)	CF27_01465 (98,61%)	PQ33_01065 (100%)	YL15_ 00560 (91,69%)
cusC-like	Acife_0052	ACH_00058 (100%)	CF27_01464 (99,23%)	PQ33_01066 (100%)	YL15_00561 (95,75%)
cusA-like	Acife_1415	ACH_01121 (100%)	CF27_01740 (99,2%)	PQ33_03116 (100%)	YL15_01694 (99,32%)
cusB-like	Acife_1416	ACH_01122 (100%)	CF27_01739 (100%)	PQ33_03117 (100%)	YL15_01693 (98,35%)
cusC-like	Acife_1417	ACH_01123 (100%)	CF27_01738 (99,8%)	PQ33_ 03118 (100%)	YL15_01692 (99,22%)
cusA-like	Acife_2127	ACH_01838 (100%)	CF27_00119 (99,51%)	PQ33_01749 (100%)	YL15_03060 (95,85%)
cusB-like	Acife_2128	ACH_01839 (100%)	CF27_00118 (100%)	PQ33_01750 (100%)	YL15_03061 (88,15%)
cusC-like	Acife_2129	ACH_01840 (99%)	CF27_00117 (98,68%)	PQ33_01751 (100%)	YL15_03062 (94,49%)
cusA-like	Acife_0198	ACH_00206 (100%)	CF27_03197 (99,51%)	PQ33_01903 (99,4%)	YL15_00377 (99,61%)
cusB-like	Acife_0199	ACH_00207 (99%)	CF27_03198 (98,67%)	PQ33_01904 (99,47%)	YL15_00378 (99,47%)
cusC-like	Acife_0200	ACH_00208 (100%)	CF27_03199 (99,58%)	PQ33_01905 (99,79%)	YL15_00379 (99,16%)
cusA-like	Acife_2138	ACH_01849 (100%)	CF27_00108 (95,85%)	PQ33_01760 (100%)	YL15_02301 (97,93%)
cusB-like	Acife_2139	ACH_01850 (100%)	CF27_00107 (98,52%)	PQ33_01761 (100%)	YL15_02302 (98,22%)
cusC-like	Acife_2140	ACH_01851 (100%)	CF27_00106 (99,61%)	PQ33_01762 (100%)	YL15_02303 (99,71%)
cusA-like	Acife_2417	ACH_02089 (100%)	CF27_03444 (99,36%)	PQ33_02561 (100%)	YL15_01608 (99,63%)
cusB-like	Acife_2418	ACH_02090 (100%)	CF27_03445 (99,44%)	PQ33_02562 (100%)	YL15_01609 (99,44%)
cusC-like	Acife_2419	ACH_02091 (100%)	CF27_03446 (99,37%)	PQ33_02563 (100%)	YL15_01610 (99,79%)
cusA-like	Acife_0822	-	CF27_03249 (85,74%)	PQ33_03198 (100%)	-
cusB-like	Acife_0823	-	CF27_03248 (58,89%)	PQ33_03199 (100%)	-
cusC-like	Acife_0824	-	CF27_03247 (66,57%)	PQ33_03200 (100%)	-
